# Disruption of iron metabolism resulting from Dmt1/Slc11a2 deficiency compromises Notch protein degradation and transcriptional activation

**DOI:** 10.1111/febs.70522

**Published:** 2026-03-30

**Authors:** Rui Zhang, Somaieh Ahmadian, Jolanda Piepers, Florian Bock, Tom Keulers, Marc A. Vooijs

**Affiliations:** ^1^ Department of Radiation Oncology (MAASTRO/GROW) Research Institute for Oncology and Reproduction Maastricht University Medical Centre The Netherlands

**Keywords:** Dmt1, ferritin, iron, iron‐response element, lipid peroxidation, LMP, lysosomes, mitochondria, NICD1, Notch, RBP‐Jκ/CSL, ROS

## Abstract

Notch receptor activation requires γ‐secretase‐mediated release of Notch intracellular domain 1 (NICD1) to regulate gene transcription. Here, we identify the proton‐driven solute carrier 11a2 (Slc11a2) or divalent metal transport protein Dmt1 as an inhibitor of Notch signaling via regulating iron homeostasis and lysosomal integrity. Dmt1 loss reduces ferritin levels and increases labile Fe^2+^, causing elevated reactive oxygen species (ROS) and lipid peroxidation. These changes compromise lysosomal function and impair degradation of S3‐Val1744 cleaved NICD1, resulting in its accumulation. Dmt1 has isoforms with or without an iron‐responsive element (IRE): Re‐expressing Dmt1 + IRE robustly increases ferritin heavy‐chain (FTH), whereas Dmt1‐IRE moderately elevates FTH and ferritin light‐chain (FTL), with co‐expression further enhancing FTL levels. Restoration of Dmt1 expression rescues ferritin levels, lysosomal activity, and NICD1 degradation while reducing oxidative stress and lipid peroxidation. Notably, Dmt1 deficiency decreases NICD1 binding to RBP‐Jκ/CSL and its recruitment to Notch target gene promoters *Hes1* and *Hey1*. Collectively, our findings demonstrate that Dmt1 regulates lysosomal function through iron homeostasis and that lysosomal dysfunction from Dmt1 loss impairs NICD1 degradation and disrupts Notch signaling, linking cellular iron metabolism and Notch pathway activity.

## Graphical abstract

Divalent metal transporter 1 (Dmt1) maintains iron homeostasis and lysosomal proteostasis required for physiological Notch receptor–ligand signaling. Dmt1 loss lowers iron storage capacity (ferritin), increasing intracellular Fe^2+^, driving ROS and lipid peroxidation, and leading to lysosomal/mitochondrial dysfunction. Despite elevated γ‐secretasecleaved NICD levels in cells and nuclei, Notch signaling fails because NICD cannot productively bind RBP‐Jκ/CSL, preventing Hes1/Hey1 transcription. Re‐expressing Dmt1 restores iron balance, organelle function, and Notch activity (created using biorender).

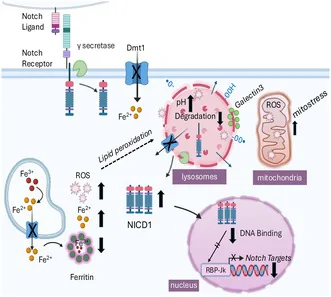

**Abbreviations**
BL1: Dmt1 inhibitor
CHX: cycloheximide
DBZ: γ‐secretase inhibitor
DCFH‐DA: dichloro‐dihydro‐fluorescein diacetate
DFO: deferoxamine
Dmt1: Divalent metal transporter1
FTH: ferritin heavy‐chain
FTL: ferritin light‐chain FTL
Gpx4: glutathione peroxidase 4
GSI: γ‐secretase inhibitor
IRE: iron‐responsive element
IRP: iron regulatory protein
Lamp1: lysosomal‐associated membrane proteins 1
LLOMe: l‐leucyl‐l‐leucine methyl ester
LMP: Lysosomal, membrane permeabilization
MEF: mouse embryonic fibroblasts
NAC: n‐acetyl cysteine
NICD: Notch intracellular domain
ROS: reactive oxygen species
Slc11a2: solute carrier family 11, member 2
TFR: transferrin receptor

## Introduction

Notch signal transduction is essential for development and cell fate in adult mammalian tissues. Activation begins with proteolytic processing of the receptor: S1‐cleavage produces a heterodimeric receptor in the Golgi; upon ligand binding, S2 cleavage by ADAM protease releases the extracellular domain, followed by S3 cleavage by the γ‐secretase complex after which Notch intracellular domain (NICD1) is released into the cytoplasm [1]. NICD1 then translocates to the nucleus, where it associates with the transcription factor RBP‐Jκ/CSL and the co‐activator MAML to activate Notch target genes.

Endocytosis of the Notch receptor in signal‐receiving cells can exert both positive and negative effects on Notch signaling [2]. Endosomal and lysosomal trafficking of both full‐length and cleaved Notch receptors is essential for Notch activation [3, 4]. This process facilitates localization of Presenilin1 and 2 [5] and enables pH‐dependent γ‐secretase cleavage [6], which together regulate NICD1 stability and transcriptional activity [7]. Notably, elevated endosomal and lysosomal pH inhibits Notch signal transduction [8, 9].

Disruption of lysosomal function by lysosomotropic agents, such as chloroquine and bafilomycin A1, has been shown to inhibit Notch‐dependent signaling downstream of γ‐secretase‐mediated cleavage [10, 11]. This indicates that γ‐secretase cleavage alone is not sufficient to drive transcriptional activation and highlights the essential role of endolysosomal trafficking in Notch signal transduction.

Previously, we identified the iron transporter Dmt1 as a novel and essential regulator of Notch signaling, demonstrating that Dmt1 isoforms serve as binary switches that control Notch‐dependent cell fate decisions in both normal and tumor cells [11, 12].

Dmt1 (Slc11a2 and Nramp2) encodes an iron transporter that mediates nonheme iron uptake in most cell types [13, 14, 15]. Its expression is tightly regulated by cellular iron levels through both translational and degradation pathways that involve iron‐responsive elements (IREs) in the 3′ UTR of Dmt1 [13, 16]. Iron homeostasis is regulated by iron regulatory proteins (IRP1 and IRP2) via the IRE pathway. IRPs bind to IRE‐containing RNA secondary structures within the untranslated region (UTR) of target mRNA, thereby modulating their translation. These include, among others, Dmt1, transferrin receptor (TFR), and the intracellular iron‐binding protein ferritin (FTH) [17, 18]. When iron levels are sufficient, iron binds to IRPs, resulting in dissociation from the IRE, promoting the translation of the target mRNA. However, under iron conditions, IRP binding to IREs inhibits translation of target mRNA [19].

Dmt1 is expressed as four different isoforms; isoforms A and B arise from alternative tissue‐specific promoters, while Isoforms I and II are with IRE (+) and without IRE (−), respectively [20, 21]. Dmt1 A isoforms are predominantly expressed in absorptive cells of the duodenum and kidney, whereas Dmt1 B isoforms are expressed ubiquitously [19, 20]. Subcellular localization also differs among isoforms: Dmt1 A‐I is primarily localized to the plasma membrane, while Dmt1 A‐II and Dmt1 B‐II are found in recycling endosomes, and Dmt1 B‐I is located at the late endosome/lysosome, respectively [21, 22]. Given the differences in expression patterns, subcellular localization of Dmt1 isoforms and effects on Notch signaling, we further investigated how Dmt1 and iron transport influence Notch‐dependent cell fate decisions.

In this study, we showed that Dmt1‐IRE expression enhances ligand‐dependent Notch signaling, while Dmt1 + IRE inhibits ligand‐dependent Notch activation [11]. Silencing of Dmt1 leads to increased lipid peroxidation and lysosomal damage. Moreover, in Dmt1‐deficient cells, we observed that intranuclear NICD1 loses its transcriptional activity due to impaired binding to the transcription factor RBP‐Jκ.

## Results

### Dmt1 loss results in increased cellular iron levels and increased ROS

**Fig. 1:**
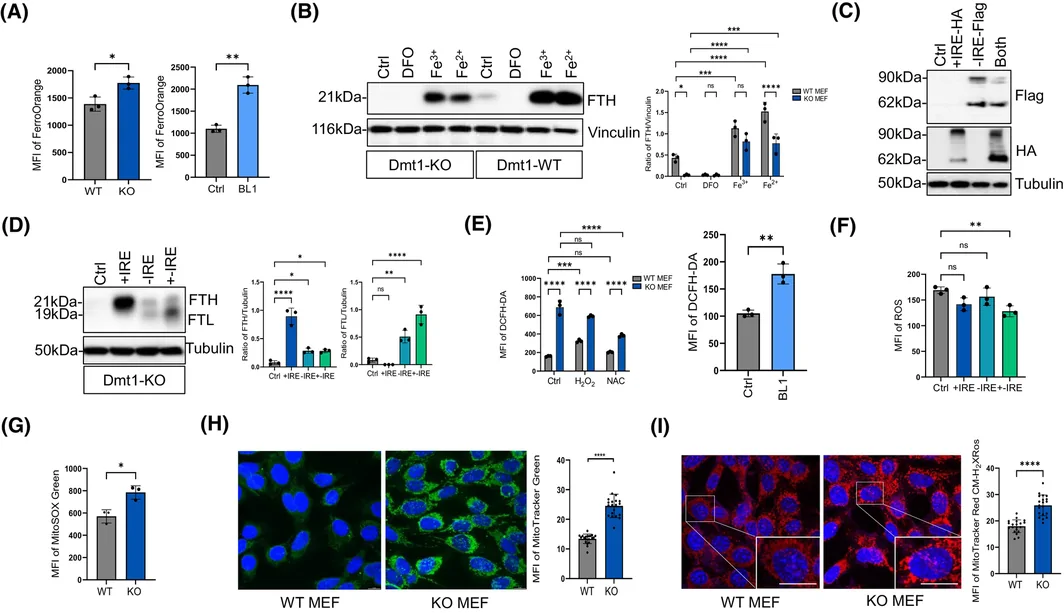
Dmt1 loss results in increased cellular iron levels and increased reactive oxygen species (ROS). (A) Quantification of intracellular Fe^2+^ levels using FerroOrange fluorescence in *Dmt*1 wild‐type (WT) and knockout (KO) mouse embryonic fibroblasts (MEFs), as well as Dmt1 WT MEFs treated by dimethyl sulfoxide (DMSO) and 1.3 μm Dmt1 inhibitor (BL1) for 24 h. (B) Expression of ferritin heavy chain (FTH) in cells treated with DMSO, 100 μm DFO for 24 h, 100 μm Fe^3+^ and Fe^2+^ for 24 h, determined by western blot. Quantification was performed by densitometry using imagej. (C) Re‐expression of Dmt1 isoforms in Dmt1 KO MEFs analyzed by western blot. (D) FTH and ferritin light‐chain (FTL) expression in Dmt1 KO MEFs reconstituted with various Dmt1 isoforms. Quantification was performed by densitometry using imagej. (E) ROS generation in Dmt1 WT and KO MEFs treated with DMSO, 100 μm H_2_O_2_ for 30 min and 10 mm NAC for 20 min, and in Dmt1 WT MEFs treated with DMSO and 1.3 μm BL1 for 24 h. ROS were measured by flow cytometry using DCFH‐DA. (F) ROS generation analysis in Dmt1 KO MEFs with the re‐expression of Dmt1 isoform via DCFH‐DA detection by flow cytometry. (G) MitoSOX Green intensity analysis in Dmt1 WT and KO MEFs by flow cytometry. (H) Representative images and quantification of MitoTracker Green staining in Dmt1 WT and KO MEFs. Nuclei were stained with 4′,6‐diamidino‐2‐phenylindole (DAPI). Scale bar: 10 μm. (I) Representative images and quantification of MitoTracker Red CM‐H_2_XRos in Dmt1 WT and KO MEFs. Nuclei were stained with DAPI. Scale bar: 10 μm. Statistical significance was assessed using an unpaired two‐tailed *t*‐test (Panels A, E, G, H, and I), one‐way ANOVA with Tukey *post hoc* tests (Panels D and F), or two‐way ANOVA with Šídák's multiple comparisons test (Panels B and E). Quantification in (H) and (I) was performed on 20 cells from independent experiments. Data are represented as mean ± standard deviation and individual dots represent independent observations. Statistical significance is indicated as *P‐value < 0.05, **P‐value < 0.01, ***P‐value < 0.001 and ****P‐value < 0.0001.

We previously established that deletion of Dmt1 in knockout (KO) mouse embryonic fibroblasts (MEF) impaired physiological Notch signaling, which correlated with increased Fe^2+^ and ROS [11]. Using FerroOrange, a fluorescent dye that binds free intracellular iron, we quantified steady‐state iron levels in Dmt1 wild‐type (WT) MEF and Dmt1 KO MEFs. In agreement with previous reports, we found that intracellular iron levels were elevated in Dmt1 KO MEF. Consistent with this, pharmacological inhibition of Dmt1 using the small molecule Dmt1 Blocker1 (BL1) reproduced the same phenotype in Dmt1 WT MEF (Fig. 1A).

To assess the impact of disrupted iron homeostasis in Dmt1 KO cells, we examined the expression of the cytosolic iron storage protein. Ferritin is composed of heavy (FTH)‐ and light chain (FTL), and its expression is tightly regulated by cytoplasmic iron availability. Dmt1 KO cells exhibited a marked reduction of FTH (Fig. 1B). Supplementing cells with exogenous ferrous Sulfate (Fe^2+^) and ferric sulfate (Fe^3+^) increased FTH levels in both WT and KO cells. As a control, incubation with the iron chelator deferoxamine (DFO) reduced ferritin expression. Importantly, re‐expression of Dmt1 isoforms fully restored FTH and FTL expression in Dmt1 KO cells (Fig. 1C,D). Notably, re‐expression of Dmt1 + IRE isoform only could rescue FTH expression, whereas re‐expression of Dmt1‐IRE rescued both FTH and FTL levels. These results show that Dm1 KO cells exhibit increased total iron levels. However, the observed decrease in ferritin expression suggests reduced availability of iron in the cytoplasm. This implies that iron may be sequestered in other cellular compartments, such as mitochondria or lysosomes, rather than being freely available in the cytosol.

Free intracellular iron pools (Fe^2+^) can generate ROS in the Fenton reaction. We therefore hypothesized that loss of Dmt1 leads to increased intracellular ROS levels. Using dichloro‐dihydro‐fluorescein diacetate (DCFH‐DA) staining of living cells, we detected an increase in steady‐state ROS levels in Dmt1 KO cells, which can be rescued by re‐expression of Dmt1 (Fig. 1E,F). Similarly, intracellular ROS levels also increased in WT MEFs treated with the Dmt1 inhibitor BL1 for 24 h (Fig. 1E). As Dmt1 loss is associated with increased cellular ROS and is localized to mitochondria, we first assessed mitochondrial superoxide levels using MitoSOX Green. A significant increase in fluorescence intensity was observed in Dmt1 KO MEFs compared with WT MEFs (Fig. 1G). We next evaluated mitochondrial staining using MitoTracker Green (MTG), as a readout of mitochondrial content/mass, and MitoTracker Red CM‐H_2_XRos, a membrane potential‐dependent mitochondrial dye. Loss of Dmt1 increased MTG fluorescence intensity (Fig. 1H), consistent with increased mitochondrial content. MitoTracker Red CM‐H_2_XRos fluorescence intensity was also increased in Dmt1 KO MEFs (Fig. 1I), consistent with increased mitochondrial membrane potential and/or mitochondrial activity. Taken together, these data indicate that loss of Dmt1 is associated with increased mitochondrial oxidative stress (MitoSOX Green), accompanied by increased mitochondrial content and increased CM‐H_2_XRos accumulation.

### ROS increases induce lipid peroxidation, associated lysosomes

**Fig. 2:**
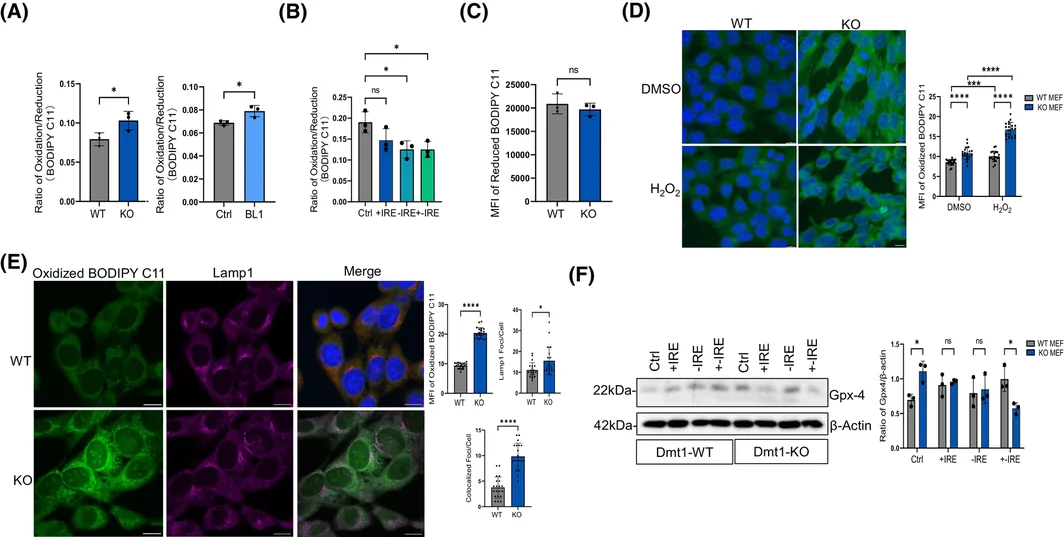
Dmt1 deficiency promotes lipid peroxidation in lysosomes. (A) Flow cytometry analysis of lipid peroxidation in Dmt1 wild‐type (WT) and knockout (KO) mouse embryonic fibroblasts (MEFs) and in Dmt1 WT MEFs treated with dimethyl sulfoxide (DMSO) and 1.3 μm BL1 for 24 h. Ratio = median Green MFI (Oxidation)/median Red MFI (Reduction) per biological replicate. (B) Lipid peroxidation analyzed by flow cytometry in Dmt1 KO MEFs with the re‐expression of Dmt1 isoforms. (C) MFI of reduced BODIPY™ 581/591 C11 (Red) in Dmt1 WT and KO MEFs analyzed by flow cytometry. (D) Representative images and quantification of oxidized BODIPY C11 (Green) in Dmt1 WT and KO MEFs treated with DMSO and 100 μm H_2_O_2_ for 30 min. Nuclei were stained with 4′,6‐diamidino‐2‐phenylindole (DAPI). Scale bar: 10 μm. (E) Representative images and quantification of oxidized BODIPY C11 (Green) colocalization with lysosomes (Lamp1, red) in Dmt1 WT and KO MEFs. Colocalization analysis was performed on 20 cells from independent experiments using imagej. Nuclei were stained with DAPI. Scale bar: 10 μm. (F) Gpx4 expression in Dmt1 WT and KO MEFs with the re‐expression of Dmt1 isoforms analyzed by western blot. Quantification was performed by densitometry using imagej. Statistical analysis was conducted using an unpaired two‐tailed *t*‐test (Panels A, C, and E), one‐way ANOVA with Tukey *post hoc* tests (Panel B), or two‐way ANOVA with Šídák's multiple comparisons test (Panels D and F). Data are represented as mean ± standard deviation, and individual dots represent independent observations. Statistical significance is indicated as *P‐value < 0.05, ***P‐value < 0.001 and ****P‐value < 0.0001.

Using electron microscopy, we previously reported that intracellular vesicles, including lysosomes in Dmt1 KO MEFs, exhibited abnormal morphology characterized by ruptured membranes and signs of permeabilization [11]. We hypothesized that this lysosomal damage may be caused by accumulated Fe^2+^ and increased ROS, resulting in lipid peroxidation of the lysosomal membrane. Using fluorescent dye BODIPY™ 581/591 C11, we observed that the ratio of Oxidation/Reduction was increased in Dmt1 KO MEFs and in WT MEF treated with Dmt1 inhibitor (Fig. 2A). Furthermore, ectopic re‐expression of Dmt1 isoforms in Dmt1 KO MEF decreased this ratio (Fig. 2B). Since the intensity of the reduced form of BODIPY C11 (581 nm) was not significantly decreased in Dmt1 KO MEFs (Fig. 2C), we focused on the oxidized BODIPY C11 signal and evaluated it using confocal microscopy. Fluorescent imaging revealed that the intensity of oxidized BODIPY C11 signal was markedly intensified by H_2_O_2_ (Fig. 2D). Importantly, the imaging showed that oxidized BODIPY C11 signal was specifically increased in the lysosomes of Dmt1 KO MEFs, indicating localized oxidative stress (Fig. 2E). This was accompanied by a rise in Lamp1‐positive foci, suggesting an increase in lysosome number. Consistent with elevated oxidative stress, glutathione peroxidase 4 (Gpx4) levels were also increased in Dmt1 KO cells, an effect that was reversed by re‐expression of the Dmt1 + ‐IRE (Fig. 2F).

### Loss of Dmt1 impairs lysosomal acidification and lysosomal intracellular activity

**Fig. 3:**
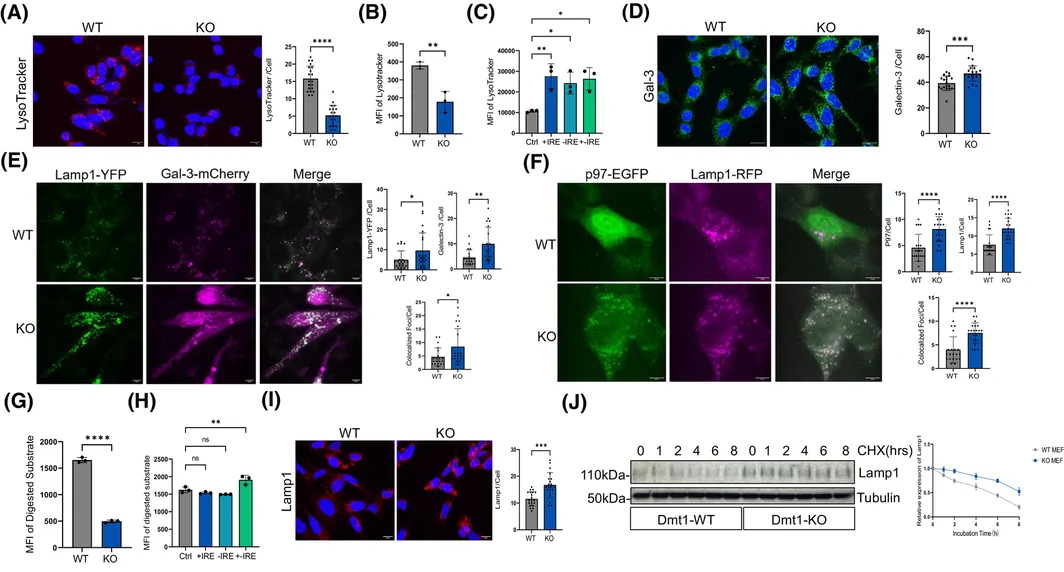
Loss of Dmt1 impairs lysosomal acidification and lysosomal intracellular activity. (A) Representative images and analysis of LysoTracker intensity in Dmt1 wild‐type (WT) and knockout (KO) mouse embryonic fibroblasts (MEFs). Twenty cells from independent experiments were quantified by imagej. Nuclei were stained with 4′,6‐diamidino‐2‐phenylindole (DAPI). Scale bar: 10 μm. (B) LysoTracker intensity analysis in Dmt1 WT and KO MEFs by flow cytometry. (C) LysoTracker intensity analysis in Dmt1 KO MEFs with the re‐expression of Dmt1 isoforms by flow cytometry. (D) Representative images and analysis of Galectin‐3 intensity in Dmt1 WT and KO MEFs. Twenty cells from independent experiments were quantified. Nuclei were stained with DAPI. Scale bar: 10 μm. (E) Representative images and quantification of colocalization between Gal‐3‐mCherry and Lamp1‐YFP in Dmt1 WT and KO MEFs. Twenty cells from independent experiments were analyzed. Nuclei were stained with DAPI. Scale bar: 10 μm. (F) Representative images and analysis of colocalization between p97‐EGFP and Lamp1‐RFP in Dmt1 WT and KO MEFs. Twenty cells from independent experiments were quantified. Nuclei were stained with DAPI. Scale bar: 10 μm. (G) Lysosomal intracellular analysis in Dmt1 WT and KO MEFs by flow cytometry. (H) Lysosomal intracellular analysis in Dmt1 KO MEFs with the re‐expression of Dmt1 isoforms by flow cytometry. (I) Representative images and quantification of Lamp1 in Dmt1 WT and KO MEFs. Twenty cells from independent experiments were quantified. Nuclei were stained with DAPI. Scale bar: 10 μm. (J) Lamp1 degradation in Dmt1 WT and KO MEFs treated with dimethyl sulfoxide (DMSO) and 50 μg·mL^−1^ cycloheximide (CHX) analyzed by western blot. The samples were collected after 0, 2, 4, 6, and 8 h of incubation with CHX and analyzed. Quantification was performed by densitometry using imagej. Statistical analysis was performed using an unpaired two‐tailed *t*‐test (Panels A, B, D, E, F, G, and I) or one‐way ANOVA with Tukey *post hoc* tests (Panel C and H). Data are represented as mean ± standard deviation, and individual dots represent independent observations. Statistical significance is indicated as *P‐value < 0.05，**P‐value < 0.01, ***P‐value < 0.001 and ****P‐value < 0.0001.

To further investigate the integrity of the lysosomal pool, we assessed the presence of intact and functional acidic lysosomes. To assess this, we used LysoTracker Red DND‐99, a lipophilic, basic fluorescent dye that selectively labels acidic organelles (pH < 5.0), such as lysosomes in living cells. Live‐cell imaging revealed a significant reduction in total integrated LysoTracker signal per cell in Dmt1 KO MEFs (Fig. 3A), consistent with decreased intensity of LysoTracker in Dmt1 KO MEFs via flow cytometry analysis (Fig. 3B) which was rescued by re‐expression of Dmt1 (Fig. 3C).

Next, we analyzed the recruitment of Galectin‐3 (Gal‐3), a well‐established marker of lysosomal membrane permeabilization (LMP). Endogenous Gal‐3 puncta were significantly increased in Dmt1 KO cells (Fig. 3D) and co‐localized with Lamp1 in cells co‐transfected with Gal‐3‐mCherry and Lamp1‐YFP (Fig. 3E), confirming lysosomal damage. Cells mitigate the harmful consequences of lysosomal leakage through autophagy‐mediated removal of damaged lysosomes. Additionally, p97 translocates to damaged lysosomes and is essential for the removal of damaged lysosomes by autophagy [23]. Consistent with this, we observed a significant increase in the colocalization of p97‐GFP and Lamp1‐RFP in Dmt1‐deficient MEFs (Fig. 3F), further confirming LMP.

Lysosomal acidification is essential for the activity of degradative enzymes like cathepsins, which require an acidic pH for substrate degradation. To assess the impact of Dmt1 deficiency on lysosomal function, we utilized flow cytometry to measure the degradation of a fluorescent lysosomal substrate reporter. Our analysis confirmed a significant impairment in substrate degradation in Dmt1‐deficient cells (Fig. 3G), which was rescued by Dmt1 re‐expression (Fig. 3H).

Furthermore, we observed an accumulation of the resident lysosomal protein Lamp1 in Dmt1 KO cells (Fig. 3I), suggesting either defective lysosomal degradation or increased protein stability. To differentiate between these, we treated Dmt1 KO cells with cycloheximide (CHX) to inhibit new protein synthesis and monitored Lamp1 turnover following CHX removal using immunoblotting. Dmt1‐deficient cells displayed increased levels of Lamp1 protein but a delayed rate of Lamp1 degradation (Fig. 3J), consistent with impaired lysosomal proteolysis.

Taken together, these data suggest that loss of Dmt1 leads to lysosomal membrane damage caused by increased lipid peroxidation, impaired lysosomal acidification, and compromised lysosomal proteolytic function.

### Dmt1 deficiency directly affects Notch degradation and transcriptional activation

**Fig. 4:**
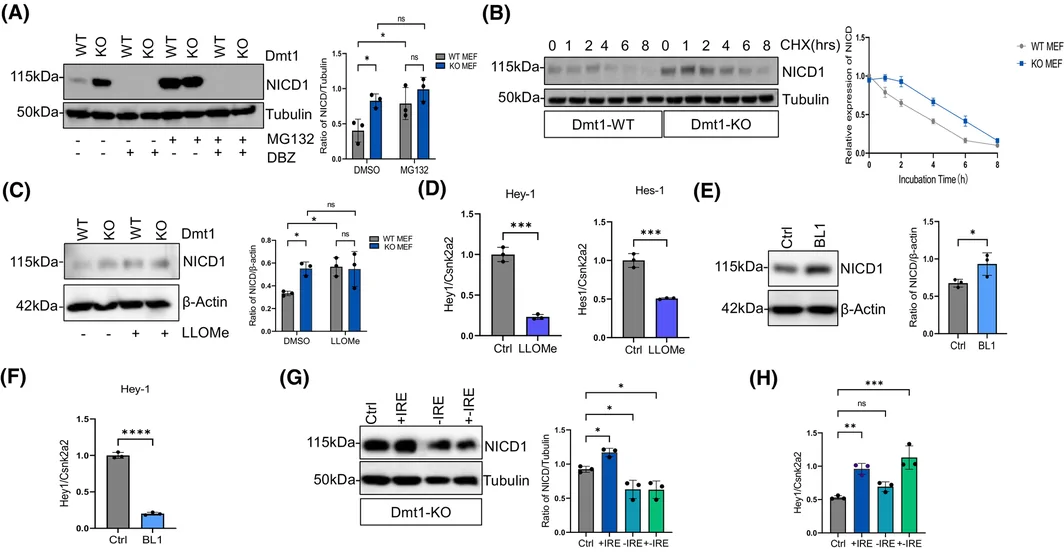
Dmt1 deficiency impairs Notch degradation and transcriptional activation. (A) NICD1 expression in Dmt1 wild‐type (WT) and knockout (KO) mouse embryonic fibroblasts (MEFs) treated with 10 μm proteasome inhibitor (MG132) for 4 h and 0.2 μm γ‐secretase inhibitor (DBZ) for 24 h analyzed by western blot. Quantification was performed by densitometry using imagej. (B) NICD1 degradation in Dmt1 WT and KO MEFs treated by dimethyl sulfoxide (DMSO) and 50 μg·mL^−1^ cycloheximide (CHX) analyzed by western blot. The samples were collected after 0, 2, 4, 6, and 8 h of incubation with CHX and analyzed. Quantification was performed by densitometry using imagej. (C) NICD1 expression in Dmt1 WT MEFs treated by DMSO and 125 μm Leu‐leu methyl ester hydrobromide (LLOMe) for 30 min analyzed by western blot. Quantification was performed by densitometry using imagej. (D) Notch target genes (*Hey1* and *Hes1*) expression in Dmt1 WT MEFs treated with DMSO and 125 μm LLOMe for 30 min analyzed by qPCR. (E) NICD1 expression in Dmt1 WT MEFs treated with DMSO and 1.3 μm BL1 for 24 h was analyzed by western blot. Quantification was performed by densitometry using imagej. (F) Notch target gene (*Hey1*) expression in Dmt1 WT MEFs treated with DMSO and 1.3 μm BL1 for 24 h, analyzed by qPCR. (G) NICD1 expression in Dmt1 KO MEFs with the re‐expression of Dmt1 isoforms analyzed by western blot. Quantification was performed by densitometry using imagej. (H) Notch target gene (*Hey1*) expression in Dmt1 KO MEFs with the re‐expression of Dmt1 isoforms analyzed by qPCR. Statistical analysis was conducted using an unpaired two‐tailed *t*‐test (Panels D–F), one‐way ANOVA with Tukey *post hoc* tests (Panels G and H), or two‐way ANOVA with Šídák's multiple comparisons test (Panel A). Data are represented as mean ± standard deviation, and individual dots represent independent observations. Statistical significance is indicated as *P‐value < 0.05, **P‐value < 0.01, ***P‐value < 0.001, and ****P‐value < 0.0001.

We previously demonstrated that Dmt1‐deficient cells exhibit defective Notch1 signaling, characterized by NICD1 stabilization without activation of Notch target genes after physiological ligand stimulation [11]. To determine whether NICD1 degradation is impaired, we examined its turnover. Notch degradation is primarily regulated by CDK8 phosphorylation and FBXW7/Cdc4‐mediated ubiquitination and proteasomal degradation [24, 25]. Treatment with the proteasome inhibitor MG132 resulted in a rapid accumulation of NICD1 within 2 h in WT cells (Fig. 4A). However, in Dmt1 KO MEFs, MG132 did not further stabilize NICD1, suggesting that proteasomal degradation of NICD1 is impaired. To directly assess NICD1 turnover, we performed pulse‐chase experiments with CHX, which revealed that NICD1 degradation was significantly delayed in Dmt1 KO cells (Fig. 4B), like the degradation defect observed for Lamp1.

To further evaluate whether an intact lysosomal pool is required for Notch signaling, lysosomes were damaged using the lysosome‐damaging agent l‐leucyl‐l‐leucine methyl ester (LLOMe). Consistent with our observation in Dmt1 KO cells, lysosomal damage induced by LLOMe resulted in NICD1 stabilization in ligand stimulated WT MEF (Fig. 4C) and reduced mRNA expression of the Notch target genes *Hey1* and *Hes*1 in WT MEFs (Fig. 4D). Additionally, pharmacological inhibition of Dmt1 with BL1 induced NICD1 stabilization (Fig. 4E) and *Hey1* mRNA suppression (Fig. 4F). Importantly, re‐expression of Dmt1 restored lysosomal substrate cleavage and reinstated NICD1 degradation (Fig. 4G), leading to recovery of Notch pathway activation, as evidenced by *Hey1* mRNA expression (Fig. 4H). Together, these results directly link Dmt1 and lysosomal function to physiological Notch1 signaling.

### Dmt1 deficiency blocks nuclear NICD1‐RBP‐Jκ/CSL complex formation and decreases recruitment of p62/SQSTM1 to NICD1

**Fig. 5:**
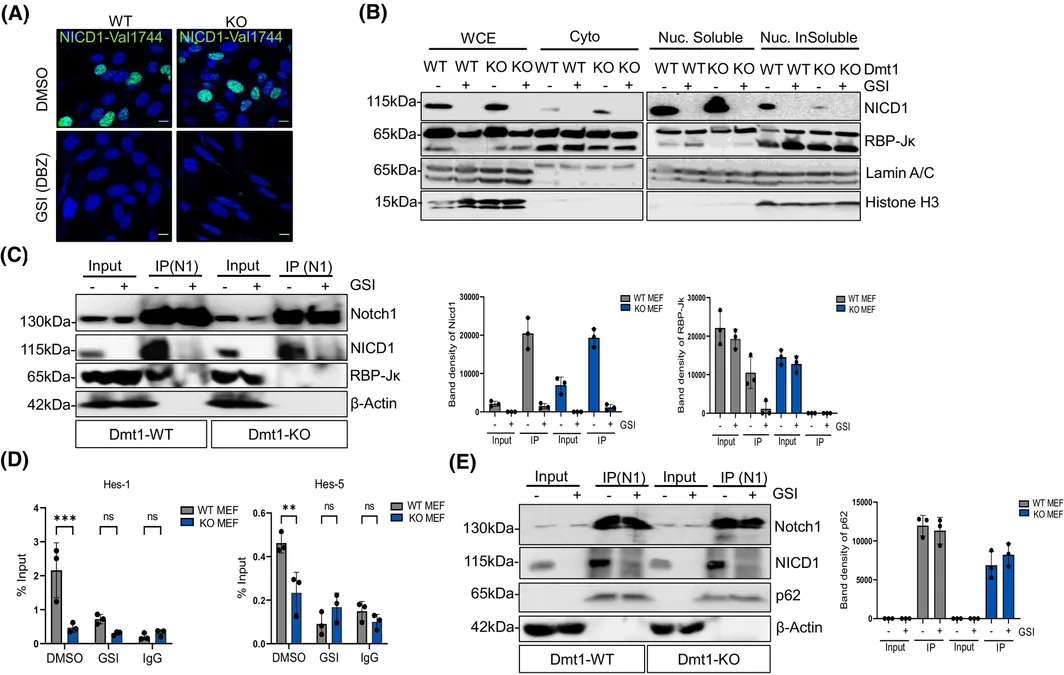
Dmt1 deficiency blocks nuclear NICD1‐RBP‐Jκ complex formation and decreases recruitment of p62 to NICD1. (A) Representative images of colocalization including NICD1 and nucleus in Dmt1 wild‐type (WT) and knockout (KO) mouse embryonic fibroblasts (MEFs) treated with dimethyl sulfoxide (DMSO) and 0.2 μm γ‐secretase inhibitor (DBZ) for 24 h. Nuclei were stained with 4′,6‐diamidino‐2‐phenylindole (DAPI). Scale bar: 10 μm. (B) NICD1 and RBP‐Jκ localization in different cell components of Dmt1 WT and KO MEFs analyzed by cell fractionation and western blot. (C) NICD1‐RBP‐Jκ complex formation analyzed by immunoprecipitation (IP) and western blot. Quantification was performed by densitometry using imagej. (D) Notch target genes (Hes1 and *Hes5*) regulation in Dmt1 WT and KO MEFs analyzed by chromatin immunoprecipitation (ChIP). (E) Recruitment of p62 to NICD1 in Dmt1 WT and KO MEFs analyzed by IP and western blot. WCE = Whole cell extract. Cyto = cytoplasm. Nuc = nucleus. Quantification was performed by densitometry using imagej. Statistical analysis was conducted using two‐way ANOVA with Šídák's multiple comparisons test (Panel D). Data are represented as mean ± standard deviation, and individual dots represent independent observations. Statistical significance is indicated as **P‐value < 0.01 and ***P‐value < 0.001.

Given the impaired Notch1 signaling observed in Dmt1‐deficient cells, we examined the subcellular localization of endogenous Notch1 following physiological ligand stimulation. Immunofluorescent staining for Val144‐cleaved NICD1 revealed nuclear accumulation in both WT and Dmt1 KO cells (Fig. 5A). Despite elevated NICD1 levels and its nuclear localization in Dmt1 KO cells, Notch signaling remained suppressed, prompting us to examine defects in the Notch transcriptional complex. To assess the NICD1 chromatin association, subcellular fractionation was performed. In Dmt1 KO cells, NICD1 was predominantly found in the soluble nuclear fraction, with reduced levels in the insoluble chromatin‐bound fraction (Fig. 5B), suggesting impaired recruitment to target gene promoters. In the canonical Notch pathway, NICD1 forms a transcriptional complex with the DNA‐binding protein RBP‐Jκ/CSL and the co‐activator Mastermind (MAML) to drive the expression of Notch target genes. RBP‐Jκ/CSL protein levels remained unchanged in Dmt1 KO cells; however, immunoprecipitation (IP) revealed a marked reduction in NICD1‐RBP‐Jκ complex formation (Fig. 5C). To determine whether this defect impaired Notch target gene regulation, we performed chromatin immunoprecipitation (ChIP). Dmt1 KO cells exhibited significantly reduced NICD1 occupancy at the *Hes*1 and *Hey*1 promoters, compared with physiological Notch signaling in WT MEFs (Fig. 5D). Furthermore, we found that the binding of the adapter p62/SQSTM1 to NICD1 was reduced in Dmt1 KO MEFs (Fig. 5E). Taken together, the results provide a direct mechanistic explanation for the reduced assembly of the transcriptional complex and activation of Notch target genes in the absence of Dmt1.

## Discussion

Our findings establish Dmt1 as a key regulator of iron homeostasis, lysosomal integrity, and physiological Notch signaling, thereby linking endogenous Notch receptor regulation to cellular iron metabolism and proteostasis [26]. Dmt1 loss leads to iron accumulation and elevated ROS, which induces oxidative stress and disrupts lysosomal function through impaired acidification and membrane permeabilization, thereby ultimately compromising proteolytic degradation and disrupting Notch signaling. Here, we show that, although present in the nucleus, the Notch intracellular domain fails to assemble with the RBP‐Jκ/CSL transcription complex required for target gene transcription, resulting in impaired Notch signaling.

In this study, we demonstrate that silencing Dmt1 impairs Notch signaling, which is associated with disruption of the endolysosomal system. In line with previous reports [11], we show that Dmt1‐deficient cells exhibit lysosomal damage, evidenced by a reduction in intact, functional lysosomes, as determined by pH‐dependent LysoTracker staining, increased membrane peroxidation, and increased Galectin‐3 localization to lysosomes. These findings indicate lysosomal membrane permeabilization, likely triggered by elevated levels of ROS caused by disrupted iron metabolism.

Dmt1 is an essential transmembrane iron transporter. Its isoforms containing or lacking an IRE (+IRE and –IRE, respectively) are localized to the plasma membrane, late endosomes/lysosomes, and recycling endosomes, where they contribute to cellular iron homeostasis. Silencing Dmt1 expression increases total cellular Fe^2+^ levels, as detected by FerroOrange, accompanied by decreased ferritin levels, the main iron storage protein. Since ferritin mRNA contains an IRE, its translation typically increases in response to elevated cytoplasmic iron. However, the paradoxical combination of elevated free Fe^2+^ and reduced ferritin in our study suggests impaired iron storage. In Dmt1‐deficient cells, impaired Fe^2+^ export causes lysosomal iron overload and loss of ferritin, thereby fueling Fenton chemistry and promoting oxidative stress, lysosomal damage, and dysfunction [27, 28].

In this reaction, Fe^2+^ catalyzes the conversion of hydrogen peroxide into highly reactive radicals which can damage cellular content, such as nucleic acids, proteins, and membranes. Notably, the increased expression of Gpx4 in KO MEFs may represent a compensatory response to ROS‐induced damage. However, the antioxidant capacity in KO MEFs was impaired, as evidenced by a significant increase in lipid peroxidation following hydrogen peroxide treatment. Further studies are needed to investigate subcellular differences in Fe^2+^ in WT and KO MEFs.

Remarkably, in addition to the high oxidative state of Dmt1 KO cells, these cells also are defective in Notch signaling. Previous studies have shown that cells with a disrupted endolysosomal system are defective in Notch signaling [8, 9, 29], highlighting the dependency of Notch signaling on the endolysosomal trafficking and function. Functional studies further demonstrated that inhibition of the lysosomal system reduces Notch signaling, in part by impairing γ‐secretase activity leading to decreased NICD availability in the nucleus [30]. Notably, genome‐wide KO screens performed in our group [11], identified Dmt1 as the most potent regulator of Notch1 signaling, ranking above other lysosome‐associated proteins. This suggests a specific and prominent role for Dmt1 and iron metabolism in regulating Notch signaling, beyond their general functions in lysosomal homeostasis [31].

Interestingly, in our study, we observed that NICD1 is still transported to the nucleus in Dmt1‐deficient cells, yet Notch target gene activation is impaired. This suggests that, beyond receptor cleavage and nuclear translocation, either (i) additional post‐translational modifications of NICD are required for its transcriptional activity, or (ii) iron is directly or indirectly required for the assembly or function of the NICD/RBP‐Jκ transcriptional complex.

One known post‐translational modification required for Notch signaling is hydroxylation, a process dependent on iron availability through iron‐dependent hydroxylases [32, 33, 34]. In line with this, the disruption of iron metabolism in our study coincides with reduced expression of Notch target genes. Therefore, one possible explanation for this finding is that iron‐dependent hydroxylases are impaired due to disrupted iron availability, thereby reducing Notch target gene activation.

Autophagy has also been implicated in the regulation of NICD stability. For instance, it has been shown that the autophagy adaptor protein p62 binds to the ubiquitinated PEST domain of NICD1, thereby promoting its degradation via autophagy [35]. Another study elucidated a potential mechanism in which the process of selective autophagic degradation of NICD1 may be initiated in the nucleus through its binding with LC3B, shown to co‐localize with the lysosomal protein Lamp1, suggesting that NICD is selectively trafficked to autolysosomes for degradation [36]. Our previous study also indicated that NICD1 is present in autophagosomes, as shown by co‐staining with LC3B [10], and it has been reported that autophagy regulates Notch degradation to modulate stem cell development [37]. Taken together with our findings, reduced p62 binding to NICD1 may contribute to the inhibition of NICD1 degradation via autophagy or proteasomal degradation.

Additionally, some histone‐modifying enzymes, such as certain histone demethylases, are iron‐dependent and require iron as a cofactor for their catalytic activity, whereas histone deacetylases (HDACs) typically depend on zinc or other metals. For instance, histone demethylase KDM5A, an iron‐dependent demethylase and essential part of the NICD/RBP‐Jκ repression mechanism [38]. Further research is needed to investigate how iron deficiency in Dmt1 KO cells affects iron‐dependent enzymes required for Notch stability and transcriptional activation.

In conclusion, our study demonstrates that Dmt1 regulates Notch signaling through its roles in iron metabolism, lysosomal integrity, and NICD1 modification. These findings reveal a mechanistic link between Dmt1 and Notch pathway activation, establishing a novel connection between iron homeostasis, lysosomal function, and Notch signaling. Given these interactions, we propose that therapeutic strategies targeting iron‐related disorders, lysosomal storage diseases, or Notch‐associated pathologies should carefully consider potential synergistic effects and unintended consequences arising from this crosstalk.

## Materials and methods

### Compounds

The reagents were used as described in the previous study [11]. Cells were treated with 100 μm Iron(II) sulfate heptahydrate (FeSO_4_·7H_2_O) (cat. no.: F7002; Sigma, Zwijndrecht, The Netherlands) for 24 h, 100 μm iron(III) sulfate hydrate (Fe_2_(SO_4_)_3_) (cat. no.: 307 718; Sigma) for 24 h, 10 mm N‐acetylcysteine (NAC) (cat. no.: A9165; Sigma) for 20 min, 100 μm hydrogen peroxide (cat. no.: H1009; Sigma) for 30 min, 0.2 μm γ‐secretase inhibitor dibenzazepine (DBZ) (cat. no.: SF4175; Syncom, Groningen, The Netherlands) for 24 h, 10 μm proteasome inhibitor MG132 (cat. no.: 474 790; Sigma) for 4 h, 100 μm deferoxamine mesylate salt (cat. no.: D9533; Sigma) for 24 h, 1.3 μm Dmt1 blocker 1 (BL1) (cat. no.: TM‐T11063; Cymit Quimica, Barcelona, Spain) for 24 h, 125 μM Leu‐leu methyl ester hydrobromide (LLOMe) (cat. no.: 16 689‐14‐8; Sigma) for 1 h and 50 μg·mL^−1^ cycloheximide (cat. no.: C7698; Sigma).

### Plasmids

pQCXIH‐Dmt1b + IRE‐HA and pQCXIH‐Dmt1b‐IRE‐Flag were constructed as described previously [11]. The following plasmids were ordered from Addgene (Watertown, MA, USA): pEGFP‐p97 (no.: 85670), Lamp1‐RFP (no.: 1817), Lamp1‐YFP (no.: 1816), and mCherry‐Gal3 (no.: 85662).

### Cell lines

Jagged2‐stimulated mNramp2 (Dmt1) WT/KO MEFs were generated as described previously [11]. Dmt1b + IRE, Dmt1b‐IRE and Dmt1 + ‐IRE re‐expression MEFs (Jagged2‐stimulated) were generated by transduction with pQCXIH‐Dmt1b + IRE‐HA, pQCXIH‐Dmt1b‐IRE‐Flag and both, respectively. Retrovirus was produced by transfecting expression vector together with packaging plasmids (pBS‐CMV‐gagpol) (cat. no.: 35614; Addgene) and envelop plasmid (pCMV‐VSV‐G) (cat. no.: 8454; Addgene) into HEK293FT cells. Cell supernatants were harvested 48 h after transfection. To obtain stable cell lines, Jagged2‐stimulated Dmt1 WT/KO MEFs were infected for 24 h with retroviral supernatants in the presence of 10 μg·mL^−1^ Polybrene (cat. no.: H9268; Sigma). Forty‐eight hours after transfection, Dmt1b + IRE re‐expression MEFs were screened with 2 mg·mL^−1^ G418 disulfate salt (cat. no.: G8168; Sigma), Dmt1b‐IRE re‐expression MEFs were screened with 1 mg·mL^−1^ hygromycin B (cat. no.: 10687010; Gibco™), and Dmt1b + ‐IRE MEFs were screened with 2 mg·mL^−1^ G418 disulfate salt and 1 mg·mL^−1^ Hygromycin B. The surviving cells were pooled and passaged before use.

All cells were maintained in DMEM (Dulbecco's Modified Eagle's Medium, high glucose) (Gibco, Amstelveen, The Netherlands) with 10% FBS under cell culture conditions (37 °C, 5% CO_2_) and regularly tested for mycoplasma contamination.

### Immunofluorescence and image processing

The procedure was conducted according to previously described methods [11]. The antibodies used are as follows: Lamp1 (cat. no.: ab24170, 1 : 1000; Abcam, Amsterdam, The Netherlands) and Galectin‐3 (cat. no.: 60207‐1‐Ig; Proteintech, Planegg‐Martinsried, Bavaria, Germany). For cells treated with fluorescent dyes, samples after fixation were counterstained with 4′,6‐diamidino‐2‐phenylindole (DAPI), mounted with mounting media, and visualized directly with confocal microscope.

For Val1744 (NICD1) staining, the immunofluorescence was performed using Alexa Fluor™ 488 Tyramide SuperBoost™ Kit, goat anti‐rabbit IgG (cat. no.: B40922; Invitrogen™, Bleiswijk, The Netherlands), based on the manufacturer's protocol. In short, MEFs were seeded in 8‐well μ‐Slides (cat. no.: 80826; ibidi, Martinsried, Bavaria, Germany) at a density of 2 × 10^4^ cells per well. Cells were fixed with 4% paraformaldehyde for 10 min, permeabilized with 0.1% Triton X‐100 for 10 min, and quenched with 50 mm glycine in PBS. Endogenous peroxidase activity was blocked using 3% hydrogen peroxide for 60 min at room temperature. Following blocking with 10% goat serum, cells were incubated with rabbit anti‐Val1744 antibody (cat. no.: 4147S, 1 : 1000; Cell Signaling, Leiden, The Netherlands) (1 : 100 in 2% BSA + 10% goat serum in 0.1% Triton X‐100) for 1 h at room temperature. After washing, cells were incubated with HRP‐conjugated goat anti‐rabbit secondary antibody for 1 h, followed by Tyramide signal amplification using Alexa Fluor 488‐conjugated Tyramide for 2–10 min. The reaction was stopped with a stop reagent, and cells were counterstained with DAPI. Cells were mounted with fluorescent mounting medium and stored at 4 °C for imaging.

Mitochondrial mass was analyzed by incubating cells with 200 nm MitoTracker Green (cat. no.: 9074S; Cell Signaling) for 30 min at 37 °C. Mitochondrial membrane potential was analyzed after incubating cells with 500 nm MitoTracker Red CM‐H_2_XRos (cat. no.: M7513; Invitrogen™) for 120 min at 37 °C. Cells were fixed with 3.7% formaldehyde in complete growth medium at 37 °C for 15 min. MitoTracker Green was imaged at excitation 488 nm and MitoTracker Red CM‐H_2_XRos at 579 nm. Images were captured with Leica SPE Confocal and processed using imagej and stored as 16‐bit TIF files.

### Immunoblotting

The procedure was carried out as previously described [11]. A total of 35–40 μg of protein was loaded per lane. Details of the primary antibodies are as follows: rabbit anti‐cleaved Notch1 (Val1744, D3B8, cat. no.: 4147S, 1 : 1000; Cell Signaling), rabbit anti‐Lamp1 (cat. no.: ab24170, 1 : 1000; Abcam), rabbit anti‐FTH (cat. no.: 3998S, 1 : 1000; Cell Signaling), mouse anti‐Vinculin (cat. no.: V9131, 1 : 1000; Sigma), mouse anti‐Tubulin (cat. no.: 2144, 1 : 1000; Cell Signaling), mouse anti‐Flag (cat. no.: F3165, 1 : 1000; Sigma), rabbit anti‐HA (cat. no.: H6908, 1 : 1000; Sigma), rabbit anti‐Gpx4 (cat. no.: 30 388‐1‐AP; Proteintech), rabbit anti‐β‐Actin (cat. no.: 81 115‐1‐RR; Proteintech), rabbit anti‐RBP‐Jκ (cat. no.: MA5‐35438; Thermo Fisher, Amsterdam, The Netherlands) for cell fractionation, rabbit anti‐Lamin A (cat.no.: L1293; Sigma), rabbit anti‐Histone H3 (cat. no.: 9715; Cell Signaling), rat anti‐Notch1 (cat. no.: 3447; Cell Signaling), mouse anti‐RBP‐Jκ (cat. no.: sc‐271 128; Santa Cruz, Heidelberg, Baden‐Württemberg, Germany) for IP, and rabbit anti‐p62 (cat. no.: 5114; Cell Signaling). Band intensity was quantified using the imagej software.

### Sample processing for flow cytometry

Iron (Fe^2+^) was detected in Dmt1 WT and KO MEFs with iron sensing dye FerroOrange (cat. no.: 36104; Cell Signaling) according to the manufacturer's protocol. Shortly, cell culture media was removed, and cells were washed with 1× PBS and subsequently incubated with 1 μmol·L^−1^ FerroOrange working solution for 30 min at 37 °C. Next, cells were washed with 1× PBS and analyzed by flow cytometry.

ROS level was analyzed by incubating cells with 5 μm 2′,7′‐dichlorofluorescin diacetate (DCFH‐DA) (cat. no.: 35845; Sigma) for 30 min at 37 °C, and cells were washed with 1× HBSS and analyzed by flow cytometry.

Mitochondrial superoxide was analyzed by incubating cells with 5 μm MitoSOX Green (cat. no.: M36005; Thermo Fisher) for 30 min at 37 °C. Next, cells were washed with 1× PBS and analyzed by flow cytometry.

Lipid peroxidation was quantified using BODIPY™ 581/591 C11 (cat. no.: D3861; Invitrogen™). Cells were incubated with 5 μm BODIPY™ C11 for 30 min at 37 °C, followed by washing with PBS. The oxidized form of BODIPY C11 was measured at 488 nm excitation and 510 nm emission, while the reduced form was detected at 581 nm excitation and 591 nm emission, respectively.

LysoTracker intensity was measured using LysoTracker Red DND‐99 (cat. no.: L7528; Invitrogen™). Cells were treated with 50 nm LysoTracker Red DND‐99 for 1 h at 37 °C and then washed with PBS. The fluorescent intensity was analyzed with an excitation and emission maximum of 577/590 nm.

Lysosomal intracellular activity was measured by Lysosomal Intracellular Activity Assay Kit (cat. no.: ab234622; Abcam) based on the manufacturer's protocol. In short, cell media was replaced with 0.5% FBS media and 15 μL substrate was added into 1 mL media. After 1 h incubation at 37 °C, cells were washed twice in 1 mL ice‐cold 1× Assay Buffer. Finally, cells were analyzed by Guava EasyCyte™ 12 flow cytometry at 488 nm excitation wavelength.

### Quantitative RT‐PCR

We performed the procedure according to methods described previously [11]. mRNA abundance was measured by the usage of forward and reverse primers (Table 1).

**Table 1:** Primers used for qPCR determination.

Target	Forward	Reverse
Csnk2a2	5′‐CCACATAGACCTAGATCCACACT‐3′	5′‐CGCAGGAGCTTGTCAAGAAGA‐3′
Hey‐1	5′‐CAGGAGGGAAAGGTTATTTTGACG‐3′	5′‐TAGTTGTTGAGATGGGAGACCAGGCG‐3′
Hes‐1	5′‐TCCTAACGCAGTGTCACCTTCCAG‐3′	5′‐CCAAGTTCGTTTTTAGTGTCCGTC‐3′
Hes‐5	5′‐ CCGACATCCTGGAGATGG‐3′	5′‐TAGCCCTCGCTGTAGTCCTG‐3′

### Cell fractionation

The fractionation protocol was described previously [39] with some adaptations. For nuclear‐cytoplasmic fractionation, MEFs were harvested by trypsinization, resuspended in complete medium to inhibit proteolysis, and centrifuged at 500× ***g*** for 4 min at 4 °C. Cell pellets were washed twice with ice‐cold PBS and centrifuged again under the same conditions. To initiate cell swelling, pellets were resuspended in hypotonic buffer (20 mm Tris–HCl pH 7.4, 10 mm KCl, 2 mm MgCl_2_, 1 mm EGTA, 0.5 mm DTT, and 0.5 mm PMSF) and incubated on ice for 3 min. NP‐40 was then added to a final concentration of 0.1%, and samples were incubated for an additional 3 min on ice. The homogenate was centrifuged at 2000× ***g*** for 5 min at 4 °C to separate the nuclear (pellet) and cytoplasmic (supernatant) fractions. The cytoplasmic supernatant was clarified by centrifugation at 15 000× ***g*** for 3 min at 4 °C to remove residual debris.

The nuclear pellet was washed two times with isotonic buffer (20 mm Tris–HCl pH 7.4, 150 mm KCl, 2 mm MgCl₂, 1 mm EGTA, 0.5 mm DTT, and 0.5 mm PMSF) then once with isotonic buffer supplemented with 0.1% NP‐40, incubated on ice for 5–10 min, and centrifuged at 1000× ***g*** for 3 min. For nuclear subfractionation, the washed nuclei were lysed in RIPA buffer (25 mm Tris–HCl pH 7.4, 150 mm NaCl, 0.1% SDS, 0.5% sodium deoxycholate, and 0.1% NP‐40, protease inhibitors). Samples were incubated on ice for 30 min and then centrifuged at 2000× ***g*** for 3 min at 4 °C to separate the RIPA‐soluble nucleosolic fraction (supernatant) from the RIPA insoluble chromatin‐bound pellet.

For further insoluble nuclear fractionation, pellets were treated with DNase I in DNase buffer (20 mm Tris–HCl pH 7.4, 100 mm NaCl, 4 mm MgCl_2_, 1 mm CaCl_2_, 0.1% NP‐40, 0.5 mm DTT, and 0.5 mm PMSF) for 30 min on ice and subsequently sonicated to release DNA‐associated proteins.

### Immunoprecipitation

Cells were lysed with ice‐cold lysis buffer (cat. no.: 87787; Thermo Scientific™) containing protease inhibitor cocktail (cat. no.: 11873580001; Roche, Rijswijk, The Netherlands). Cell lysates were centrifuged at 14 000 ***g*** for 15 min at 4 °C, and the supernatant was collected for concentration test by Bradford (cat. no.: 5000001; Bio‐Rad, Munich, Bavaria, Germany). Protein was mixed with Notch1 antibody (cat. no.: 3608S; Cell Signaling) at 4 °C overnight. Protein G beads (cat. no.: 10003D; Invitrogen™) were immunoprecipitated with protein and antibody complex at 4 °C 1 h. The beads were washed 3 times with lysis buffer and then heated at 100 °C in SDS sample buffer for immunoblotting.

### Chromatin immunoprecipitation

Sample preparation was performed as described previously [40]. Specifically, the chromatin pull‐down antibody and beads were the same as IP. Normal Rabbit IgG antibody (cat. no.: 2729S; Cell Signaling) was used as the control. The sequences of oligonucleotides used as ChIP primers are listed in Table 1.

### Statistical analysis

Data were presented as mean ± SD with three biological repeats. Statistical analyses were performed using graphpad prism 9. The unpaired two‐tailed *t*‐test was used for single comparisons, one‐way ANOVA with Tukey *post hoc* tests was used for intergroup comparisons, and two‐way ANOVA with Šídák's multiple comparisons test was used for comparison across multiple experimental groups. Results were considered significant if *P‐value < 0.05, **P‐value < 0.01, ***P‐value < 0.001 and ****p‐value < 0.0001.

## Author contributions

RZ, SA, and JP performed the experiments and analyzed the experimental data. RZ drafted the manuscript and designed the figures under the supervision of FB, TK, and MAV. FB, TK, and MAV contributed to the design and implementation of the research. MAV supervised the study. All authors discussed the results and commented on the manuscript.

## Conflict of interest

The authors declare no conflict of interest.

## Data Availability

The datasets generated during the current study are available in the Figshare repository at https://doi.org/10.6084/m9.figshare.30933263.
